# Sweetpotato bZIP Transcription Factor *IbABF4* Confers Tolerance to Multiple Abiotic Stresses

**DOI:** 10.3389/fpls.2019.00630

**Published:** 2019-05-16

**Authors:** Wenbin Wang, Xiangpo Qiu, Yanxin Yang, Ho Soo Kim, Xiaoyun Jia, Huan Yu, Sang-Soo Kwak

**Affiliations:** ^1^College of Life Science, Shanxi Agricultural University, Taigu, China; ^2^College of Arts and Science, Shanxi Agricultural University, Taigu, China; ^3^Plant Systems Engineering Research Center, Korea Research Institute of Bioscience and Biotechnology, Daejeon, South Korea

**Keywords:** abiotic stress, drought tolerance, salt tolerance, *IbABF4*, sweetpotato

## Abstract

The abscisic acid (ABA)-responsive element binding factors (ABFs) play important regulatory roles in multiple abiotic stresses responses. However, information on the stress tolerance functions of *ABF* genes in sweetpotato (*Ipomoea batatas* [L.] Lam) remains limited. In the present study, we isolated and functionally characterized the sweetpotato *IbABF4* gene, which encodes an abiotic stress-inducible basic leucine zipper (bZIP) transcription factor. Sequence analysis showed that the IbABF4 protein contains a typical bZIP domain and five conserved Ser/Thr kinase phosphorylation sites (RXXS/T). The *IbABF4* gene was constitutively expressed in leaf, petiole, stem, and root, with the highest expression in storage root body. Expression of *IbABF4* was induced by ABA and several environmental stresses including drought, salt, and heat shock. The IbABF4 protein localized to the nucleus, exhibited transcriptional activation activity, and showed binding to the *cis*-acting ABA-responsive element (ABRE) *in vitro*. Overexpression of *IbABF4* in *Arabidopsis thaliana* not only increased ABA sensitivity but also enhanced drought and salt stress tolerance. Furthermore, transgenic sweetpotato plants (hereafter referred to as SA plants) overexpressing *IbABF4*, generated in this study, exhibited increased tolerance to drought, salt, and oxidative stresses on the whole plant level. This phenotype was associated with higher photosynthetic efficiency and lower malondialdehyde and hydrogen peroxide content. Levels of endogenous ABA content and ABA/stress-responsive gene expression were significantly upregulated in transgenic *Arabidopsis* and sweetpotato plants compared with wild-type plants under drought stress. Our results suggest that the expression of *IbABF4* in *Arabidopsis* and sweetpotato enhances tolerance to multiple abiotic stresses through the ABA signaling pathway.

## Introduction

Abiotic stresses such as drought, salinity, and high temperature severely affect plant growth, development, and productivity (Moore et al., 2009). Understanding the mechanisms of abiotic stress tolerance in plants is a crucial topic of environmental research (Bartels and Sunkar, 2005). In response to adverse environment conditions, plants have evolved a number of defense mechanisms involving transcription factors (TFs) that bind to conserved *cis*-acting elements in target gene promoters, thus activating their expression and leading to enhanced stress tolerance (Fujita et al., 2005). Numerous stress-responsive TFs, such as those belonging to the basic leucine zipper (bZIP), WRKY, MYB, basic helix-loop-helix (bHLH), and NAC families, have been well-characterized through genetic, molecular, and biochemical analyses (Wang et al., 2003; Golldack et al., 2011). Transgenic plants overexpressing these TFs show improved tolerance to different environmental stresses (Hossain M. et al., 2010).

The bZIP proteins, containing a basic DNA-binding region and a leucine zipper dimerization motif, comprise one of the largest and diverse TF families (Latchman, 1993). Recent studies show that bZIP TFs are involved in diverse biological processes such as abiotic and biotic stress responses, seed germination, flower and seed development, and hormone and sugar signaling (Thurow et al., 2005; Muszynski et al., 2006; Lindemose et al., 2013). In *Arabidopsis thaliana*, the bZIP TFs are classified into 10 groups, based on sequence similarity of the basic region and additional conserved motifs (Corrêa et al., 2008). The abscisic acid (ABA)-responsive element (ABRE)-binding factors (ABFs), also known as ABA-responsive element binding (AREB) proteins, belong to Group A of the bZIP protein family. The consensus core sequence (C/T)ACGTGGC has been identified as a major *cis*-acting regulatory element (Hirayama and Shinozaki, 2010; Yoshida et al., 2010). The ABFs regulate the expression of ABA and other stress-responsive genes by binding to the ABREs in their promoter regions (Busk and Pagès, 1998; Hyungin et al., 2000). Five conserved Ser/Thr kinase phosphorylation sites (RXXS/T) are characteristic of the abiotic stress-responsive *AtABFs/AREB* proteins. Amino-acid residue (S26, S86, S94, T135) of four RXXS/T phosphorylation target sites in the N-terminal three conserved regions can be phosphorylated by SnRK2-type protein kinases and further activate AREB1 protein (Furihata et al., 2006; Fujii et al., 2009). Furihata et al. (2006) also confirmed that additional kinases exist for the phosphorylation of AREB1b in response to salt and/or osmotic stresses. The *ABF* genes have been extensively investigated in a number of plant species, such as *Arabidopsis*, rice (*Oryza sativa*), wheat (*Triticum aestivum*), barley (*Hordeum vulgare*), potato (*Solanum tuberosum*), and tomato (*Solanum lycopersicum*) (Hyungin et al., 2000; Casaretto and Ho, 2005; Kobayashi et al., 2008; Hossain M.A. et al., 2010; Tsaihung et al., 2010; García et al., 2012). Most *ABF* genes such as *ABF2/AREB1*, *ABF4/AREB2*, and *ABF3* are highly induced by ABA, drought, and salt treatments (Uno et al., 2000; Furihata et al., 2006; Yoshida et al., 2010). Furthermore, overexpression of *ABFs* increases abiotic stress tolerance in several plant species (Hossain M.A. et al., 2010; Roychoudhury et al., 2013). Transgenic *Arabidopsis* plants overexpressing *ABF3* and *ABF4* exhibit improved drought tolerance via the up-regulation of several ABA and other stress-responsive genes (Kang et al., 2002). Overexpressing *AtABF3* in alfalfa (*Medicago sativa*) reduces the transpiration rate and accumulation of reactive oxygen species, and increases tolerance to drought, salt, and oxidative stresses (Wang Z. et al., 2016).

Sweetpotato (*Ipomoea batatas* [L.] Lam) is the seventh most important food crop on the basis of the annual starch production worldwide (Pradhan et al., 2015), and has the potential to be commercially utilized as a health food as it is rich in antioxidants, dietary fiber, and minerals. Furthermore, sweetpotato is widely used as a source of starch and bioethanol (Madzlan et al., 2012; Duvernay et al., 2013). However, pests, viral diseases, and various environmental stresses, such as drought and extreme temperature, generally limit the production of sweetpotato in many areas worldwide (Lebot, 2010). Therefore, understanding the mechanisms of adaptation to adverse environmental conditions in sweetpotato plants is key for the development of stress tolerant cultivars.

In this study, we isolated the sweetpotato *IbABF4* gene, which encodes an abiotic stress-inducible bZIP TF, and characterized its expression in different tissues and in response to ABA and several environmental stresses. The transactivation and ABRE-binding ability of IbABF4 was tested using a yeast one-hybrid assay and electrophoretic mobility shift assays (EMSAs). We also generated transgenic *Arabidopsis* and sweetpotato plants overexpressing *IbABF4*. The tolerance of these transgenic plants to drought, salt, and oxidative stresses was investigated at the seed and whole plant levels. Our results indicate that *IbABF4* is a positive transcriptional regulator of the abiotic stress response, and overexpression of *IbABF4* significantly increases the tolerance of transgenic plants to multiple abiotic stresses.

## Materials and Methods

### Plants Materials and Growth Conditions

Sweetpotato cultivar Xushu 18 and *Arabidopsis thaliana* ecotype Colombia-0 (Col-0) were used in this study. Col-0 plants, used as the wild-type (WT), were grown in a growth chamber at 22 ± 1°C under 16 h light/8 h dark photoperiod. Sweetpotato plants were propagated by cuttings, which were grown at 25 ± 1°C under a 16 h light/8 h dark photoperiod. To analyze gene expression, 3-week-old sweetpotato plants were cultured in half-strength Hoagland nutrient solution supplemented with 10 μM ABA, 25% polyethylene glycol (PEG) 8000 (drought stress), 350 mM NaCl (salt stress), respectively, and subjected to 47°C (heat stress) treatments. The third leaf from the top of the plants was sampled at 0, 3, 6, 12, 24, and 48 h for further quantitative real-time PCR analysis. Plant tissues (leaves, petioles, stems, fibrous roots, pencil roots, proximal end of storage roots, storage root bodes, and distal end of storage roots) were separated from the 10-week-old sweetpotato plants under no environmental stress for tissue-specific gene expression analysis.

### Gene Isolation and Plasmid Construction

Total RNA of sweetpotato cultivar Xushu18 was used for cDNA synthesis. Full-length cDNA of the *IbABF4* gene was isolated by reverse transcription PCR (RT-PCR) using sequence-specific primers (Supplementary Table S1) and cloned into the pGWB5 vector using the gateway method (Curtis and Grossniklaus, 2003). In this vector, the *IbABF4* gene was fused to the *green fluorescent protein* (*GFP*) gene under the control of the cauliflower mosaic virus (CaMV) *35S* promoter. The resulting *35S::IbABF4-GFP* plasmid was used for subcellular localization analysis and *Agrobacterium*-mediated transformation.

To determine the transcriptional activation activity of *IbABF4*, full-length or partial (N- or C-terminal) coding sequences (Figure 3B) of *IbABF4* cDNA were cloned in the pDEST32 vector containing the GAL4 DNA-binding domain (pBD) using the gateway method. This generated three in-frame protein fusion constructs, *pBD::IbABF4*, *pBD::IbABF4ΔN*, and *pBD::IbABF4ΔC*.

To conduct EMSAs, full-length *IbABF4* cDNA was cloned into the pDEST15 vector containing the glutathione *S*-transferase (GST) tag using the gateway method, thus generating the *GST::IbABF4* plasmid.

### Quantitative Real-Time PCR (qRT-PCR) Analysis of Gene Expression

Total RNA and cDNA templates were obtained as described previously (Jin et al., 2017). All qRT-PCR analyses were performed in triplicate on a CFX Connect Real-Time PCR Detection system (Bio-Rad, Berkeley, CA, United States) using gene-specific primers (Supplementary Table S1) and Ever-Green PCR master mix kit (BioFact, Daejeon, South Korea), according to the manufacturer’s instructions. Relative expression levels of genes were calculated using the 2^–ΔΔCT^ method.

### Subcellular Localization of IbABF4

The fusion construct *35S::IbABF4-GFP* was transformed into *Agrobacterium tumefaciens* strain GV3101, which was then infiltrated into tobacco (*Nicotiana benthamiana*) leaves to obtain transient expression of IbABF4. After 3 days of culture, the infiltrated parts of leaves were cut and immersed in 4′,6-diamidino-2-phenylindole (DAPI) solution for nuclear staining and subjected to fluorescent signal detection under a confocal laser scanning microscope (Leica Microsystems, Heidelberg, Germany) with proper filter sets (Ke et al., 2017).

### Transcriptional Activation Activity of IbABF4

Three fusion constructs *pBD::IbABF4*, *pBD::IbABF4ΔN*, and *pBD::IbABF4ΔC*, as well as pBD (pDEST32 empty vector; negative control) and pGAL4 (positive control) vectors were transformed individually into the yeast strain PJ69-4A containing *His3* and *LacZ* reporter genes. The transformed yeast cells were cultured on synthetic defined (SD) plates containing media lacking either only leucine (SD/Leu-) or Leu and histidine (SD/Leu-/His-). The transactivation activity of each protein was evaluated by the filter lift assay and quantitative assays of β-galactosidase activity using *O*-nitrophenyl-β-d-galactopyranoside (ONPG) as a substrate. All procedures were performed according to the Yeast Protocols Handbook (Clontech, United States).

### Recombinant Protein Purification and EMSAs

The GST::IbABF4 fusion protein was expressed in *Escherichia coli* strain BL21 and purified as described previously (Jin et al., 2017). Optimal conditions for protein expression were created by adding 0.4 mM isopropyl-β-d-thiogalactoside (IPTG) and by incubating the *E. coli* cells at 20°C for 12 h. Oligonucleotide sets containing the ABRE repeat motif (5′-GGACA GCTGGC GGGACACGTGGC GGGACACGTGGC G-3′) were annealed by boiling for 5 min and then labeled with [γ-^32^P]-ATP by adding T4 Polynucleotide Kinase (Promega, Madison, WI, United States). A 30 μL mixture of a labeled probe (0.5 μg) and purified GST-IbABF4 or GST protein (10 μg) was incubated in binding buffer (200 mM HEPES, 5 mM DTT, 1 mM EDTA, 50 mM KCl, and 20 pmol of poly dI-dC) at room temperature for 30 min. Subsequently, the reaction mixture was loaded on an 8% native polyacrylamide gel and visualized by autoradiography. Competition experiments were carried out by incubating increasing amounts of unlabeled competitor probe with purified GST-IbABF4 fusion protein before the addition of labeled probes.

### Generation of Transgenic Sweetpotato and *Arabidopsis* Plants Overexpressing *IbABF4*

To overexpress the *IbABF4* gene in sweetpotato cultivar Xushu 18, *IbABF4* expression plasmids were introduced into *A. tumefaciens* strain EHA105 using the freeze-thaw method (Höfgen and Willmitzer, 1988) and then transformed into embryogenic calli of Xushu 18 plants via *Agrobacterium*-mediated transformation, as described previously (Lim et al., 2004; Kang et al., 2017). The transformed calli were selected on Murashige and Skoog (MS) medium containing 400 mg L^–1^ cefotaxime and 25 mg L^–1^ hygromycin. To generate transgenic *Arabidopsis* plants overexpressing *IbABF4*, the *35S::IbABF4-GFP* recombinant plasmid was introduced in *A. tumefaciens* strain GV3101 and then transformed into Col-0 plants by the floral dip method (Clough and Bent, 1998). Homozygous T3 lines were used for further analysis.

Transgenic sweetpotato and *Arabidopsis* lines were confirmed and selected by genotyping and qRT-PCR analysis of hygromycin resistant plants. Primers used for PCR and qRT-PCR analyses are listed in Supplementary Table S1.

### Stress Tolerance Assay

To perform cotyledon greening analyses, surface-sterilized Col-0 and T3 seeds were sown on half-strength MS (1/2 MS) medium supplemented with or without 0.6 μM ABA, 200 mM mannitol, or 125 mM NaCl. Greening of seedlings was determined at 1 week post-germination. To perform root growth assays, 5-days-old *Arabidopsis* seedlings were transferred to 1/2 MS medium containing 300 mM mannitol or 125 mM NaCl to induce drought or salt stress, respectively. Root length and fresh weight were measured after 10 days. To determine the drought tolerance of adult plants, irrigation of 3-week-old plants was withheld for 10 days, followed by re-watering. After the plants were photographed, contents of malondialdehyde (MDA), ABA and hydrogen peroxide (H_2_O_2_) and expression levels of stress-responsive genes were measured. Gene-specific primer sequences are listed in Supplementary Table S1.

To assess the stress tolerance of sweetpotato plants, 5-week-old plants of the WT and two transgenic lines were subjected to drought (without irrigation for 17 days and recovery for 2 days) or salt (irrigation with 200 mM NaCl solution every 3 days for 6 days) stress treatments. After photographing the plants, the third leaf from the top of the plants was used for the analysis of photosynthetic efficiency (*Fv/Fm*) of photosystem II (PSII) and measurement of ABA, MDA, and H_2_O_2_ contents. In plants subjected to drought stress, ABA content (at 3 and 6 days) and expression pattern of stress-responsive marker genes (at 6 days) were also measured. To determine the effect of oxidative stress on transgenic sweetpotato (hereafter referred to as SA) and WT plants, leaf disks (1 cm diameter) were excised from the third or fourth healthy leaf from the top of 5-week-old plants and submerged in 5 mL medium containing 2 μM methyl viologen (MV). The mixture was incubated in the dark for 12 h, followed by continuous light at 25°C. The analysis of relative membrane permeability (at 0, 12, 24, 36, and 48 h) and 3,3′-diaminobenzidine (DAB) staining (at 48 h) were performed according to the methods described by Jin et al. (2017) and Kang et al. (2017), respectively. All experiments were repeated three times.

### Measurement of *Fv/Fm* and ABA, MDA, and H_2_O_2_ Contents

The *Fv/Fm* values were measured using a portable chlorophyll fluorescence meter (Handy pEA, Hansatech, England) after 30 min of dark adaptation. The ABA content of SA and WT plants was measured using an enzyme-linked immunosorbent assay (ELISA), as described previously (Yang et al., 2001). The MDA content of plants was measured using the modified thiobarbituric acid (TBA) method described by Wang et al. (2009). Values of specific and non-specific absorbance of extracts were measured at 532 and 600 nm, respectively. Non-specific absorbance was then subtracted from the specific absorbance. The H_2_O_2_ content of plants was assessed using the xylenol orange method, as described previously (Bindschedler et al., 2001; Wang et al., 2009). The H_2_O_2_ content was expressed as μmol of H_2_O_2_ per gram of fresh weight of plant tissue.

### Statistical Analysis

All experimental assays were repeated at least three times. Significant differences between means were determined by analysis of variance (ANOVA and Tukey–Kramer test) at *P* < 0.01.

## Results

### Isolation and Structural Analysis of *IbABF4*

**FIGURE 1:**
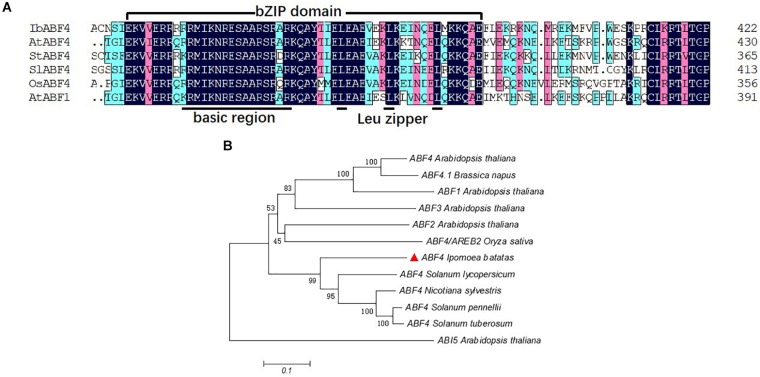
Multiple sequence alignment of the highly conserved bZIP domains and phylogenetic analysis of selected ABF-related proteins. **(A)** Multiple sequence alignment of the conserved bZIP domains of selected ABF-related proteins. Color-coding indicates sequence similarity, with black indicating the highest sequence similarity, pink indicating lower similarity, and blue indicating the least similarity. **(B)** Phylogenetic analysis of IbABF4 homologs in plants.

The full-length cDNA sequence of *IbABF4* isolated from Xushu 18 was 1,272 bp in length, with an open reading frame of 423 amino acids (GenBank Accession No. MK503986). Analysis of the deduced amino acid residues using the SMART program^1^ revealed that the IbABF4 protein contained a typical bZIP domain (N-X7-R/K-X9-L-X6-L-X6-L), including a basic DNA-binding domain and a leucine zipper domain (Figure 1A). Five conserved Ser/Thr kinase phosphorylation sites (RXXS/T) were also observed in IbABF4 (data not shown). Furthermore, alignment and phylogenetic analysis revealed that IbABF4 was the most closely related to the ABF4 homologs of tomato and *Nicotiana sylvestris* (Figure 1B).

### Tissue-Specific and Stress-Induced Expression Patterns of *IbABF4*

**FIGURE 2:**
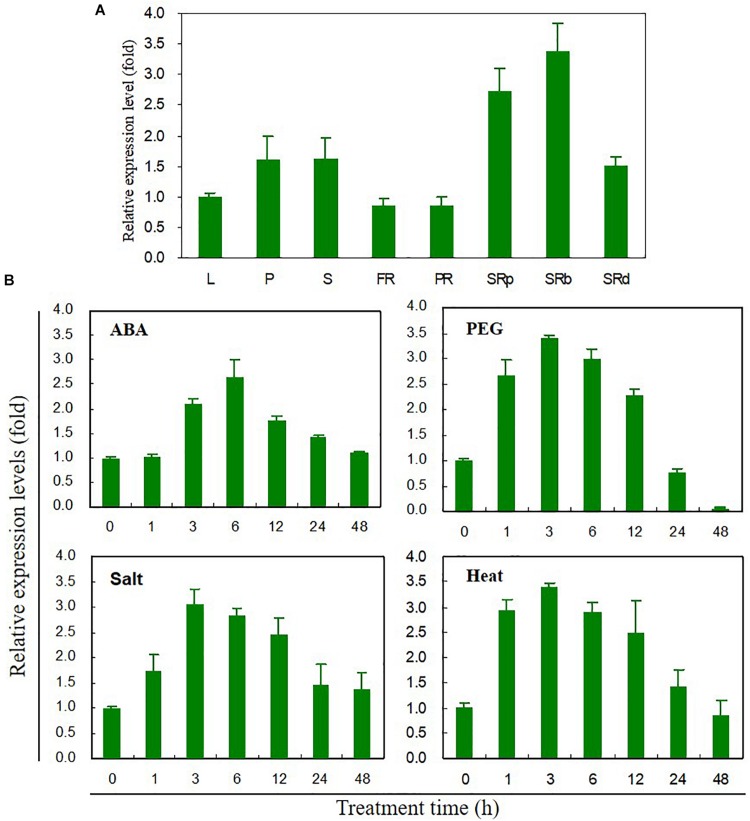
Expression profiles of *IbABF4* in various tissues and under various abiotic stress conditions. **(A)** Tissue-specific expression pattern of *IbABF4*. L, leaf; P, petiole; S, stem; FR, fibrous root; PR, pencil root; SRp, proximal end of storage root; SRb, storage root body; SRd, distal end of storage root. **(B)** Expression pattern of *IbABF4* in response to various abiotic stresses. Three-week-old sweetpotato plants grown in half-strength Hoagland nutrient solution were subjected to ABA (10 μM), dehydration (25% PEG8000), high salt (350 mM NaCl), and heat shock (47°C) treatments. Expression levels of genes were determined in the third fully expanded intact leaf (from the top) by qRT-PCR analysis. Expression levels of genes were normalized relative to the *IbActin* gene (internal control). Data represent mean ± standard deviation (SD) of three biological replicates.

Tissue-specific expression levels of *IbABF4* were measured by qRT-PCR (Figure 2A). *IbABF4* was expressed in all analyzed tissues, with higher expression in the storage root, especially storage root body, compared with other tissues. The relative expression level of *IbABF4* in sweetpotato plants exposed to ABA, PEG, NaCl, and heat was also measured by qRT-PCR (Figure 2B). In plants exposed to 10 μM ABA, the relative expression level of *IbABF4* started increasing at 3 h, reaching a peak at 6 h, and then declining over the following 12 h. At the peak time point, the expression of *IbABF4* in ABA-treated plants was 2.7-fold higher than that in untreated control plants. The expression of *IbABF4* was also strongly induced by 25% PEG, 350 mM NaCl, and 47°C heat shock treatments, with expression patterns similar to those observed in ABA-treated plants. Under dehydration, salinity, and heat stresses, the expression of *IbABF4* was the highest at 3 h; at this time point, *IbABF4* expression in stress-treated plants was 3.5-, 3-, and 3.5-fold higher than that in control plants, respectively. Taken together, these results suggest that *IbABF4* is induced by ABA, dehydration, salinity, and high temperature.

### IbABF4 Localizes to the Nucleus and Exhibits Transcriptional Activation Capability

**FIGURE 3:**
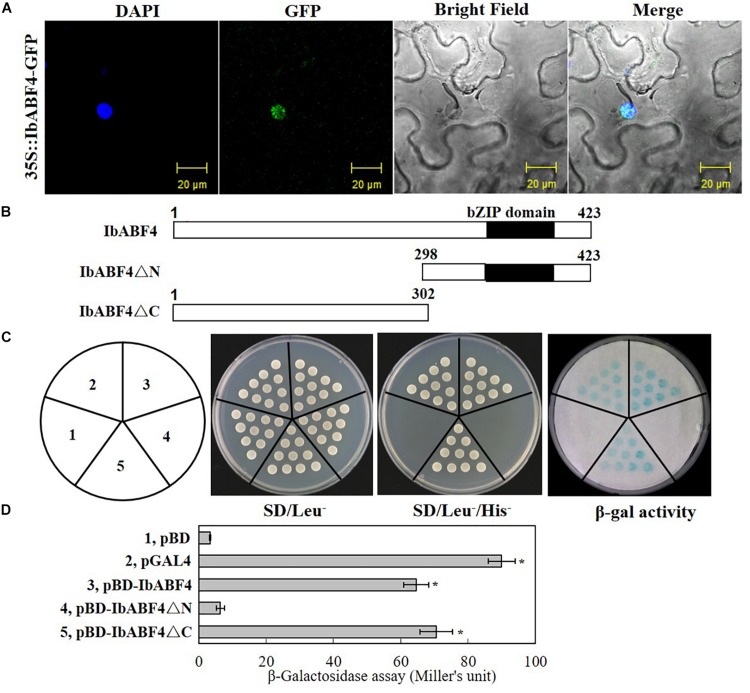
Subcellular localization and transactivation assay of the IbABF4 protein. **(A)** Subcellular localization of IbABF4. **(B)** Vectors used in the yeast one-hybrid assay. **(C)** Transactivation activity assay of the IbABF4 protein. The pGAL4 and pBD empty vectors were used as positive and negative controls, respectively, in yeast one-hybrid assay. **(D)** Relative quantitative assay of β-galactosidase activity (expressed in Miller units). Data represent mean ± SD of three independent biological replicates. Asterisks indicate significant differences between pBD and other vectors at *P* < 0.01.

We determined the subcellular localization of IbABF4 by transiently expressing the *35S::IbABF4-GFP* plasmid in tobacco leaf epidermal cells. Co-localization of green fluorescence with the DAPI signal indicated that the IbABF4 protein was localized to the nucleus (Figure 3A).

We further determined the transcriptional activation capability of IbABF4 protein using the yeast one-hybrid assay. Full-length and N- and C-terminal fragments (IbABF4ΔC and IbABF4ΔN, respectively) of the IbABF4 protein were cloned into the yeast expression vector pBD containing the GAL4 DNA BD (Figure 3B). The pGAL4 and pBD empty vectors were used as positive and negative controls, respectively. All transformants grew well on SD/Leu- plates (Figure 3C). Yeast cells harboring pGAL4, *pBD::IbABF4*, and *pBD::IbABF4ΔC* grew normally on SD/Leu-/His- medium and showed β-galactosidase activity, whereas yeast cells containing pBD and *pBD::IbABF4ΔN* failed to grow on the SD/Leu-/His- medium and did not show β-galactosidase activity. These results revealed that IbABF4 possesses transcriptional activation capability, dependent on its N-terminal bZIP region. The results of the relative quantitative assay of β-galactosidase activity, using ONPG as a substrate, were consistent with those of the yeast one-hybrid assay (Figure 3D).

### IbABF4 Protein Specifically Binds to the *cis*-Acting ABRE *in vitro*

**FIGURE 4:**
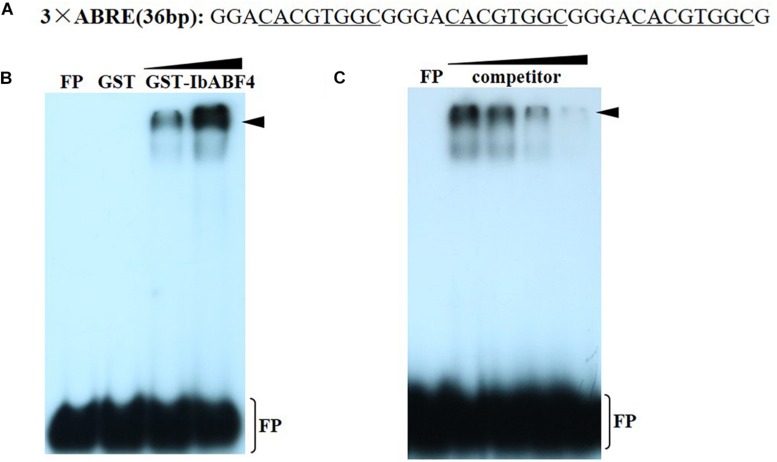
Binding of IbABF4 to the *cis*-acting ABRE in EMSA. **(A)** Oligonucleotide probes containing the ABRE repeat motif. The underlined letters indicate the ABRE core sequence. **(B)** Analysis of binding specificity of IbABF4. FP indicates free probe. EMSA was performed using ^32^P-labeled ABRE probe with GST protein (lane 2) or GST-IbABF4. Triangles indicate increasing amounts of GST-IbABF4 protein (5 and 10 μg) used for DNA-binding analysis. **(C)** Competition analysis of unlabeled probe in EMSA. Triangles indicate increasing amounts of unlabeled probe. GST-IbABF4 protein was preincubated with 1-, 20-, 40-, and 80-fold molar excess of ABRE before the addition of probe.

The *cis*-acting ABRE, which is often present in the promoters of ABA and other stress-responsive genes, is the binding site of the ABF/AREB proteins. To determine whether IbABF4 binds to the *cis*-acting ABRE *in vitro*, we performed EMSA using recombinant GST-IbABF4 fusion protein and ^32^P-labeled DNA containing three repeats of the ABRE core sequence (Figure 4A). Expression of GST-IbABF4 in *E. coli* BL21 cells was induced by the addition of IPTG, and the recombinant fusion protein was successfully purified by affinity chromatography. Results of the EMSA showed a shift in electrophoretic mobility of the ^32^P-labeled ABRE probe (Figure 4B). The binding activity of GST-IbABF4 increased with an increase in the amount of protein (Figure 4B, lanes 3 and 4). By contrast, GST alone did not bind to the ^32^P-labeled ABRE probe (Figure 4B, lane 2). The addition of increasing amounts of unlabeled ABRE probe prior to the addition of labeled probes (Figure 4C, lanes 3, 4, and 5) gradually abolished the binding. These results indicate that IbABF4 specifically binds to the *cis*-acting ABRE *in vitro*.

### IbABF4 Affects ABA Sensitivity and Stress Tolerance Levels of Transgenic *Arabidopsis* Seeds

**FIGURE 5:**
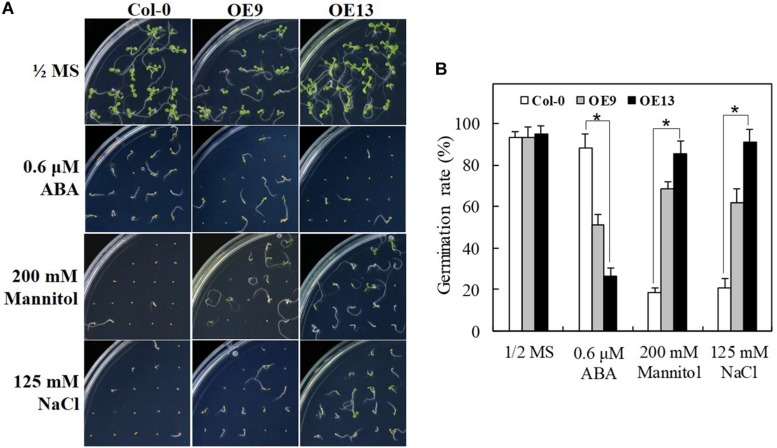
Seed germination assay of transgenic *Arabidopsis* lines overexpressing *IbABF4* under ABA, drought, and salt stresses. **(A)** Seed germination phenotype of transgenic and WT Col-0 seeds on 1/2 MS medium supplemented with or without (control) 0.6 μM ABA, 200 mM mannitol, or 125 mM NaCl. **(B)** Germination rate of transgenic and Col-0 seeds under ABA, drought, and salt stresses. A total of 100 seeds were used for the calculation of germination rate in each treatment. Data represent mean ± SD of three biological replicates. Asterisks indicate significant differences at *P* < 0.01.

To characterize the function of *IbABF4* in ABA, drought, and salt stress tolerance during seed germination, two independent T3 homozygous lines (OE9 and OE13) of *Arabidopsis* overexpressing *IbABF4* were selected (data not shown) for further analysis; WT Col-0 plants were used as a control. Under normal growth conditions, no differences were observed in seed germination phenotype (Figure 5A) and germination rate (Figure 5B) between OE and WT plants. However, OE seeds were more sensitive to exogenously applied ABA than WT seeds during germination. The OE seeds showed significant improvement in germination rate upon exposure to mannitol and NaCl compared with WT seeds. These results suggest that *IbABF4* increases the level of ABA sensitivity and drought and salt stress tolerance in plants.

### Overexpression of *IbABF4* Promotes Root Elongation in *Arabidopsis* Seedlings Under Drought and Salt Stresses

**FIGURE 6:**
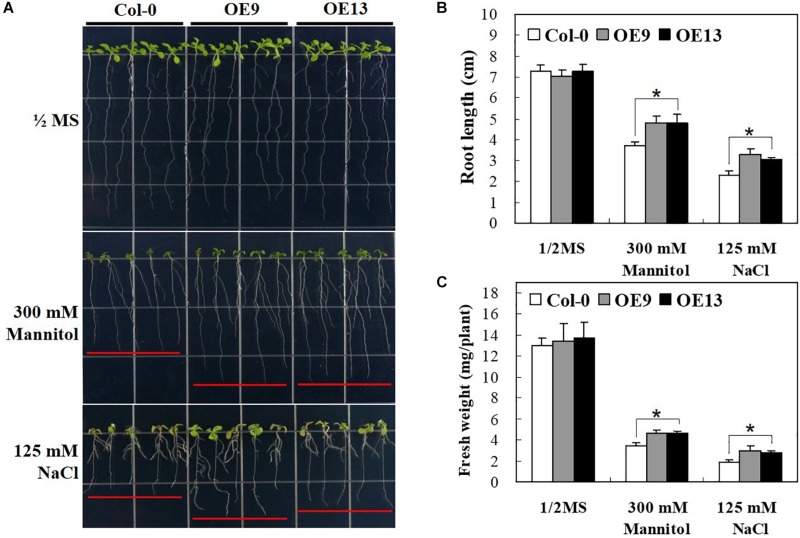
Post-germination growth assay of transgenic *Arabidopsis* plants overexpressing *IbABF4* under drought and salt stresses. **(A)** Growth of transgenic *Arabidopsis* plants on 1/2 MS medium supplemented with or without (control) 300 mM mannitol, or 125 mM NaCl. **(B)** Root length of *Arabidopsis* plants subjected to drought and salt stresses. **(C)** Fresh weight of *Arabidopsis* plants exposed to drought and salt stresses. A total of 40 seedlings were used in each treatment. Data represent mean ± SD of three biological replicates. Asterisks indicate significant differences at *P* < 0.01.

To gain further insights into the possible roles of *IbABF4* in abiotic stress tolerance at post-germination, we investigated the growth phenotype of transgenic *Arabidopsis* seedlings overexpressing *IbABF4* under drought and salt stresses. Under normal growth conditions, no difference was observed in growth phenotype (Figure 6A), root length (Figure 6B), and fresh weight (Figure 6C) between OE plants and WT. By contrast, under drought and salt stresses imposed by 1/2 MS medium containing 300 mM mannitol or 125 mM NaCl, respectively, OE plants exhibited significantly greater root elongation and fresh weight than WT plants. In the presence of 300 mM mannitol, root length was 4.7 cm in OE9 and OE13 lines but only 3.7 cm in WT seedlings. These results indicate that constitutive overexpression of *IbABF4* enhances drought and salt tolerance of transgenic *Arabidopsis* plants.

### Overexpression of *IbABF4* Increases Drought Stress Tolerance at the Whole Plant Level in *Arabidopsis*

**FIGURE 7:**
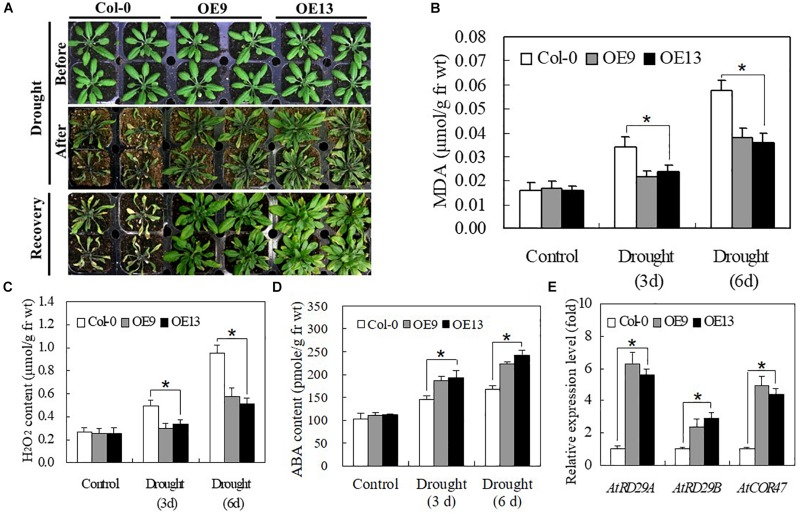
Overexpression of *IbABF4* in *Arabidopsis* confers drought tolerance. **(A)** Drought tolerance phenotypes of 3-week-old transgenic *Arabidopsis* (OE9 and OE13) lines and Col-0 plants grown in soil without irrigation for 10 days, followed by recovery for 1 day. Contents of MDA **(B)**, H_2_O_2_ **(C)** and ABA **(D)** in leaves of Col-0 and OE plants grown without irrigation for 3 and 6 days. **(E)** Transcript levels of stress-responsive marker genes in OE9, OE13, and Col-0 plants. The *AtActin* gene was used as an internal control. Data represent mean ± SD of three biological replicates. Asterisks indicate significant differences at *P* < 0.01.

We evaluated the drought tolerance of transgenic and WT *Arabidopsis* plants grown in soil. No significant differences were detected between transgenic and WT plants under normal growth conditions. However, when irrigation was withheld for 10 days, most of the leaves of WT plants turned yellow, shrunk in size, and ultimately died. By contrast, plants of transgenic *Arabidopsis* lines OE9 and OE13 only displayed slight wilting but grew well and recovered successfully following re-watering for 1 day (Figure 7A). The MDA and H_2_O_2_ contents of both transgenic and WT plants gradually increased at 3 and 6 days of drought treatment, although transgenic lines exhibited lower MDA and H_2_O_2_ contents than WT plants (Figures 7B,C). The ABA content of OE plants was higher than that of WT plants at 3 and 6 days of drought stress (Figure 7D). Furthermore, the expression of stress-responsive genes, including *AtRD29A*, *AtRD29B*, and *AtCOR47*, was highly induced by drought stress, with significantly higher expression in transgenic *Arabidopsis* plants than in WT Col-0 plants (Figure 7E). Taken together, these data suggest that IbABF4 is a functional homolog of *Arabidopsis* ABF4, which activates stress-responsive genes such as *AtRD29A*, *AtRD29B*, and *AtCOR47*, thus enhancing drought tolerance.

### Overexpression of *IbABF4* in Sweetpotato Increases Tolerance to Drought Stress

**FIGURE 8:**
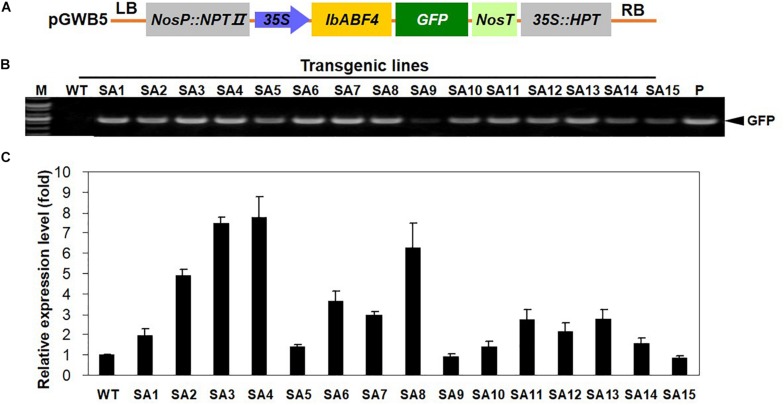
Generation of SA plants overexpressing *IbABF4*. **(A)** Schematic representation of the constructs used for sweetpotato transformation. **(B)** PCR analysis of SA plants using *GFP*-specific primers. M, DNA size markers; P, positive control. **(C)** qRT-PCR analysis of SA plants using *IbABF4*-specific primers. The *IbActin* gene was used as an internal control. Data represent mean ± SD of three biological replicates.

The results of transgenic *Arabidopsis* plants encouraged us to generate transgenic sweetpotato plants with improved abiotic stress tolerance through the overexpression of *IbABF4*. The *IbABF4* cDNA was cloned in the pGWB5 vector under the control of CaMV *35S* promoter (Figure 8A), and transgenic sweetpotato plants were generated by *Agrobacterium*-mediated transformation. A total of 15 independent hygromycin resistant sweetpotato lines were confirmed by PCR amplification of genomic DNA (Figure 8B). Two transgenic lines (SA3 and SA4) exhibiting high *IbABF4* expression were identified by qRT-PCR for further characterization (Figure 8C).

**FIGURE 9:**
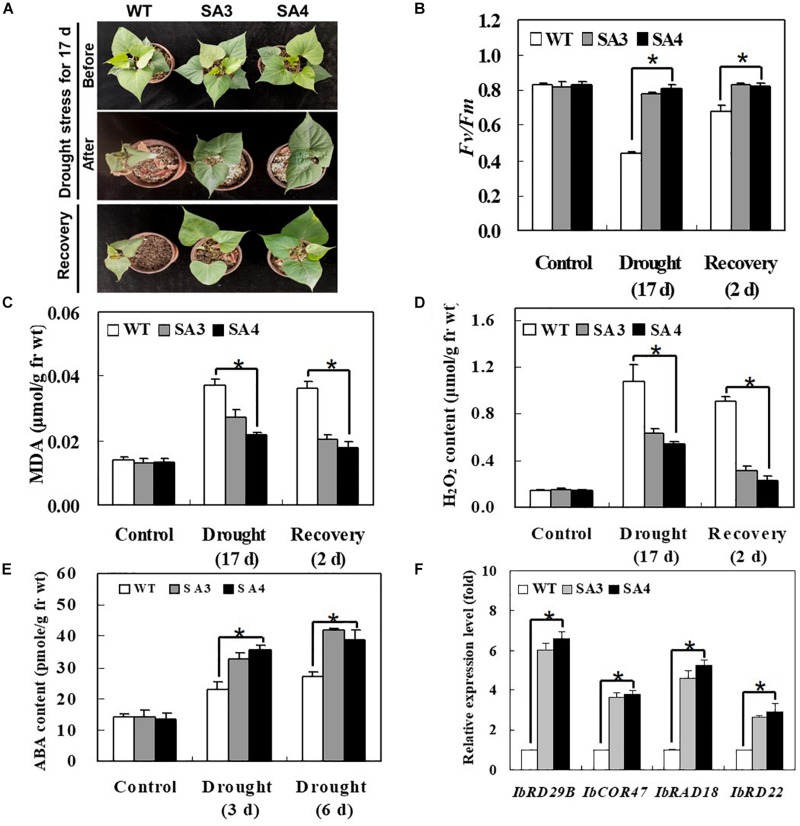
Phenotypic and physiological characterization of SA plants under drought stress. **(A)** Visible damage in the leaves of sweetpotato plants after drought stress treatment for 17 days, followed by recovery for 2 days. **(B)** Photosynthetic efficiency (*Fv/Fm*) of PSII. Contents of MDA **(C)** and H_2_O_2_ **(D)** in leaves of 5-week-old WT and SA plants subjected to drought stress for 17 days, followed by recovery for 2 days. **(E)** ABA content of leaves of 5-week-old WT and SA plants subjected to drought stress for 3 and 6 days. **(F)** Transcript levels of stress-responsive marker genes in WT and SA plants determined by qRT-PCR. Samples of WT plants were collected prior to drought stress treatment, and transcript levels of genes were normalized relative to *IbActin* transcripts. Data represent mean ± SD of three biological replicates. Asterisks indicate significant differences between WT and SA plants at *P* < 0.01.

To evaluate the effect of *IbABF4* overexpression in sweetpotato on drought stress tolerance, we compared the growth phenotypes of SA and WT plants subjected to water deficiency for 17 days, followed by recovery for 2 days. Under drought stress conditions, SA plants showed slight wilting but grew well and recovered successfully, whereas WT plants displayed severe wilting at 17 days and slight recovery after re-watering for 2 days (Figure 9A). The SA plants maintained higher photosynthetic efficiency (*Fv/Fm*) of PSII throughout the water deficiency and recovery periods than WT plants (Figure 9B). In both WT and SA plants, MDA and H_2_O_2_ contents increased after 17 days of continuous drought stress and then decreased after 2 days of recovery. The WT plants consistently exhibited a higher level of MDA and H_2_O_2_ contents than SA plants throughout the drought stress and recovery periods (Figures 9C,D). Taken together, these results suggest that *IbABF4* overexpression in sweetpotato enhances drought tolerance.

We also examined the content of ABA content and expression pattern of several stress-responsive marker genes in SA and WT plants subjected to drought stress. The ABA content of SA plants was higher than that of WT plants at 3 and 6 days of drought stress (Figure 9E). The SA plants also exhibited significantly higher expression levels of ABA/stress-responsive marker genes, including *IbRD29B*, *IbCOR47*, *IbRAB18*, and *IbRD22*, than WT plants (Figure 9F). Increased levels of ABA content and stress-responsive marker gene expression in SA plants likely explain their enhanced drought tolerance.

### Overexpression of *IbABF4* in Sweetpotato Increases Tolerance to Salt Stress and MV-Induced Oxidative Stress

**FIGURE 10:**
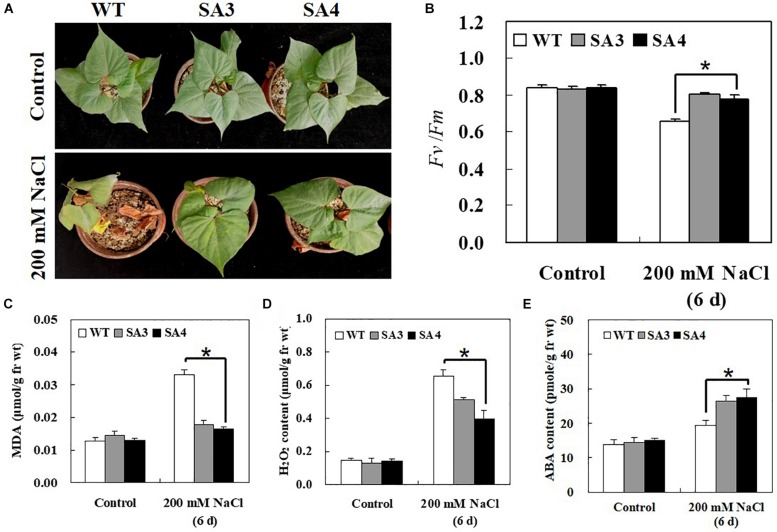
Phenotypic and physiological characterization of SA plants under salt stress. **(A)** Visible damage in the leaves of sweetpotato plants after 6 days of salt stress treatment. **(B)** PSII photosynthetic efficiency (*Fv/Fm*). Contents of MDA **(C)**, H_2_O_2_ **(D)**, and ABA **(E)** in leaves of 5-week-old WT and SA plants subjected to salt stress treatment for 6 days. Data represent mean ± SD of three biological replicates. Asterisks indicate significant differences between WT and SA plants at *P* < 0.01.

To determine whether *IbABF4* overexpression in sweetpotato confers salt stress tolerance, 5-week-old WT and SA plants were irrigated with 200 mM NaCl solution every 3 days for 6 days. Under normal growth conditions, no phenotypic differences were observed between WT and SA plants. However, under salt stress, WT plants exhibited more severe wilting and chlorosis than SA plants (Figure 10A). The SA plants also showed higher *Fv/Fm* values than WT plants at 6 days of salt stress treatment (Figure 10B). Furthermore, under salt stress conditions, contents of MDA and H_2_O_2_ in WT plants were significantly higher than those in SA plants (Figures 10C,D). The ABA content of SA plants was higher than that of WT plants at 6 days of salt stress (Figure 10E).

**FIGURE 11:**
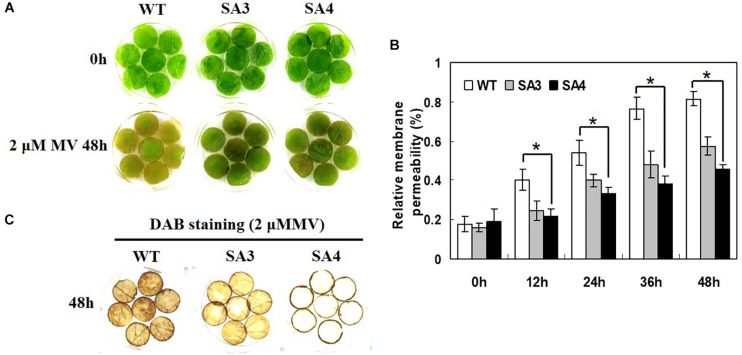
Oxidative stress tolerance of SA plants. **(A)** Visible damage in detached leaves of WT and SA plants treated with 2 μM MV. **(B)** Relative membrane permeability of detached leaves treated with 2 μM MV at 48 h. Data represent mean ± SD of three biological replicates. Asterisks indicate significant differences between WT and SA plants at *P* < 0.01. **(C)** Analysis of H_2_O_2_ accumulation in detached leaves using DAB staining. The third or fourth fully expanded leaves were collected from the top of 5-week-old WT and SA plants.

A leaf disk senescence assay was performed to investigate the oxidative stress tolerance of SA plants. When treated with MV, WT plants showed symptoms of injury including rapid senescence and chlorosis, whereas SA plants only exhibited slight damage (Figure 11A). Moreover, SA plants showed significantly lower relative membrane permeability than WT plants (Figure 11B). We also investigated the level of H_2_O_2_ accumulation following MV-induced oxidative stress by DAB staining. Detached leaves of SA plants exhibited reduced DAB staining intensity than those of WT plants after MV treatment (Figure 11C), indicating lower levels of H_2_O_2_ in SA leaves. Overall, we conclude that overexpression of *IbABF4* confers tolerance to salt stress and MV-induced oxidative stress in sweetpotato.

## Discussion

Sweetpotato is one of the most important food crops, as it is rich in nutrients and useful as a source of starch and bioethanol (Duvernay et al., 2013; Pradhan et al., 2015). The production of sweetpotato is limited by a variety of abiotic stresses, resulting in significant yield losses (Lebot, 2010). Therefore, the expression and function of genes regulating abiotic stress response in sweetpotato are of considerable interest. Several sweetpotato genes including *IbOr* (Kim et al., 2013; Goo et al., 2015; Kang et al., 2017), *IbCBF3* (Jin et al., 2017), *IbPsbP* (Kang et al., 2017), *IbCHY-β* (Kang et al., 2017), *IbLCY-β* (Kim et al., 2014), *IbMPK3*, and *IbMPK6* (Kim et al., 2016) have been cloned and characterized in our laboratory. Although many genes encoding bZIP TFs are known to play key roles in abiotic stress response, only a few such genes have been reported in sweetpotato. In this study, we demonstrated that a Group A bZIP encoding gene, *IbABF4*/*IbABRE2*, is a component of the ABA signaling pathway involved in adaptation to drought, salt, and oxidative stresses.

Based on the basic DNA-binding domain containing an invariant motif (N-X7-R/K) and a leucine zipper domain (L-X6-L-X6-L), bZIP TFs are classified into 10 groups in *Arabidopsis* (Jakoby et al., 2002) and 13 groups in rice (Corrêa et al., 2008). The ABF/AREB proteins belong to Group A of bZIP TFs. Five conserved Ser/Thr kinase phosphorylation sites (RXXS/T) are characteristic of the abiotic stress-responsive *AtAREB/ABFs* (Furihata et al., 2006; Fujii et al., 2009). In the present study, we showed that like other ABFs, IbABF4 contains the typical bZIP domain (Figure 1A) and five conserved Ser/Thr kinase phosphorylation sites. Amino acid sequence alignments demonstrated that IbABF4 is similar to other ABFs including AtABF4, OsABF4, StABF4, SlABF4, and AtABF1. Together with phylogenetic analysis (Figure 1B), these findings suggest that IbABF4 is a functional homolog of plant ABF4 proteins responsive to abiotic stresses.

The expression of several ABA/stress-responsive genes in plants is regulated by *cis*-acting elements that function as important molecular switches in the transcriptional regulation of a dynamic network of genes (Yamaguchishinozaki and Shinozaki, 2005, 2006). Subcellular localization experiments in this study indicated that IbABF4 functions as a transcriptional regulator in the nucleus (Figure 3A). The results of yeast one-hybrid assay revealed that IbABF4 possesses transcriptional activation capability dependent on its N-terminal bZIP domain (Figures 3C,D). Our results are consistent with those of previous yeast one-hybrid assays of OsABF1 (Hossain M. et al., 2010), OsABF2 (Hossain M.A. et al., 2010), OsbZIP23 (Xiang et al., 2008), and TaAREB3 (Wang J. et al., 2016). Furthermore, the results of EMSAs indicate that IbABF4 specifically binds to the *cis*-acting ABRE with the core sequence CACGTGGC *in vitro* (Figure 4). Similar observations on *AtABF1*, *SlAREB*, *TabZIP60*, *TaAREB3*, and *TabZIP14-B* are reported in yeast one-hybrid assay and EMSAs (Hyungin et al., 2000; Tsaihung et al., 2010; Zhang et al., 2015, 2017; Wang J. et al., 2016). In conclusion, these data suggest that IbABF4 is a transcriptional activator that specifically binds to the *cis*-acting ABRE in the promoters of downstream stress-responsive genes, thus activating their expression.

Most *ABF* genes such as *ABF2/AREB1*, *ABF4/AREB2*, and *ABF3* are highly induced by ABA, drought, and salinity treatments in plant tissues (Uno et al., 2000; Furihata et al., 2006; Yoshida et al., 2010). Overexpression of *ABFs* increases abiotic stress tolerance in several plant species (Roychoudhury et al., 2013). In this study, transcript levels and response times of *IbABF4* to ABA, PEG, salt, and heat shock treatments indicated that *IbABF4* is highly sensitive to environmental stresses (Figure 2B). Thus, our data indicate that *IbABF4* plays an important role in the response to abiotic stresses. Compared with WT Col-0 seeds, transgenic *Arabidopsis* seeds overexpressing *IbABF4* exhibited a significantly lower germination rate when treated with exogenous ABA and an improved germination rate when treated with mannitol or NaCl (Figure 5). Additionally, *Arabidopsis* OE plants exhibited significantly greater root length and fresh weight than Col-0 plants under drought and salt stress treatments (Figure 6). Furthermore, lower MDA and H_2_O_2_ contents of transgenic *Arabidopsis* plants than WT plants indicate that transgenic plants have greater tolerance to salt and dehydration stresses (Figure 7). Moreover, we successfully generated SA plants overexpressing *IbABF4* (Figure 8). Under drought and salt stresses, SA plants exhibited lower MDA and H_2_O_2_ contents than WT plants (Figures 9C,D, 10C,D), suggesting that *IbABF4* overexpression increases antioxidant activity in sweetpotato. The reduced *Fv/Fm* values of WT plants indicated that the PSII reaction center was more severely damaged in WT plants than in SA plants (Figures 9B, 10B). Under MV-induced oxidative stress, SA plants showed only slight damage compared with WT plants (Figure 11). Together, these results suggest that *IbABF4* overexpression improves the germination rate and post-germination growth of transgenic lines under drought, salt, and oxidative stress conditions. Our results are consistent with a previous study of Kim et al. (2004) showing that *Arabidopsis abf3* and *abf4* mutants display defects in response to ABA, salt, and dehydration stresses.

In the present study, endogenous ABA contents were higher in transgenic plants than in WT plants under drought stress (Figures 7D, 9E). Moreover, transcript levels of several stress-responsive genes including *RD29A*, *RD29B*, *COR47*, *RAB18*, and *RD22* were upregulated in transgenic *Arabidopsis* and sweetpotato plants overexpressing *IbABF4* under drought stress (Figures 7E, 9F). All stress-responsive genes harbor ABREs in their promoter regions (Fujita et al., 2005; Yamaguchishinozaki and Shinozaki, 2006). These results support our conclusion that *IbABF4* mediates responses to drought stress via the ABA signaling pathway. The results of EMSA suggest that *IbABF4* interacts with ABREs in the promoters of ABA-responsive genes, thus enhancing the expression level of these genes and resulting in greater drought tolerance (Agarwal and Jha, 2010). These genes are also induced by salt stress and ABA (Nakashima et al., 2009; Tsaihung et al., 2010). Increased expression levels of these genes may, at least in part, explain the improved drought and salt stress tolerance of transgenic plants. There is evidence for cross-talk between ABA signals and H_2_O_2_ signaling in plant tissue (Saxena et al., 2016; Phillips and Ludidi, 2017). In present study, transgenic *Arabidopsis* and sweetpotato plants overexpressing *IbABF4* showed increasing ABA and H_2_O_2_ contents under drought and salt treatments than normal growth condition (Figures 7C,D, 9D,E, 10D,E). We deduced that H_2_O_2_ may function as an intermediate in ABA signaling. MAPK cascade is activated by the increasing H_2_O_2_ which in turn is mediated by the hormones like ABA (Saxena et al., 2016). Subsequently, ABA-activated SnRK2-type protein kinases may phosphorylate the Ser/Thr residues of RXXS/T sites and activate IbABF4 protein similar to AtAREB1 (Furihata et al., 2006). The lower level of H_2_O_2_ contents in transgenic *Arabidopsis* and sweetpotato plants than WT under stresses treatments (Figures 7C, 9D, 10D) may be caused by over expression of various downstream antioxidants such as CAT and APX mediated by IbABF4 protein. However, the ABA and H_2_O_2_ signaling pathways in IbABF4 protein response to abiotic stress still need further investigation. The higher level of H_2_O_2_ contents in WT plants caused corresponding increases in lipid peroxidation (Figures 7B, 9C, 10C).

## Conclusion

We report that the *IbABF4* TF specifically binds to the *cis*-regulatory ABRE. Overexpression of *IbABF4* in *Arabidopsis* resulted in enhanced tolerance to drought and salt stresses. Moreover, SA plants confirmed the role of *IbABF4* in tolerance to drought, salt, and oxidative stresses. Agronomic traits of sweetpotato plants overexpressing *IbABF4* need further characterization in the field. Overall, the results of this study broaden our understanding of sweetpotato bZIP TFs and suggest an excellent candidate gene for the improvement of stress tolerance in crop plants.

## Data Availability

All datasets generated for this study are included in the manuscript and/or the Supplementary Files.

## Author Contributions

WW, HK, and S-SK conceived and designed the experiments. WW, XQ, YY, HK, XJ, and HY performed the experiments. WW, XQ, YY, and HK analyzed the data. S-SK contributed to reagents, materials, and analysis tools. WW, XQ, and S-SK wrote the manuscript .

## Conflict of Interest Statement

The authors declare that the research was conducted in the absence of any commercial or financial relationships that could be construed as a potential conflict of interest.

## Footnotes

1 http://smart.embl-heidelberg.de
