# Supplementary material for: Sweetpotato bZIP Transcription Factor IbABF4 Confers Tolerance to Multiple Abiotic Stresses
Source: Front Plant Sci. 2019 May 16;10:630. doi: 10.3389/fpls.2019.00630 (PMC6531819; doi:10.3389/fpls.2019.00630)
Supplement: Supplementary file 1 [file Table_1.DOCX]

| **Primer name** | **Primer sequence (5’ to 3’)** | **Application** |
| --- | --- | --- |
| *IbABF4-F* | ATGATGGGGTCATACTTGGA | Vector construction |
| *IbABF4-R* | TTACCAAGGCCCAGTAAGCG | Vector construction |
| *GFP-F* | AGGTTATTGGAAAATTAAGGGCC | Genomic DNA PCR |
| *GFP-R* | AACCAGATCCGATTTTGGAGGATG | Genomic DNA PCR |
| *IbABF4-RT-F* | CAGCTGAATAGTATGGGATT | Real-time PCR |
| *IbABF4-RT-R* | CTGGTGGCTCCTACACTAAG | Real-time PCR |
| *AtRD29A-F* | GCCGAGAAACTTCAGATTGG | Real-time PCR |
| *AtRD29A-R* | CCATTCCTCCTCCTCCTTTC | Real-time PCR |
| *AtRD29B-F* | CCGCAAAGAACGTCGTTGCCTCA | Real-time PCR |
| *AtRD29B-R* | CCACCTCCGGAGAGAGGTAGCT | Real-time PCR |
| *AtCOR47-F* | GCATGACCATCCCGAGGAAGAG | Real-time PCR |
| *AtCOR47-R* | ACTTCCTCTTCAGTGGTCTTGGC | Real-time PCR |
| *IbRD29B-F* | TCAAGGACACGCTTACCCAC | Real-time PCR |
| *IbRD29B-R* | GGCTTCTCCACAGGACCAAA | Real-time PCR |
| *IbCOR47-F* | AAACGCTCCACCGCTCCTCTA | Real-time PCR |
| *IbCOR47-R* | CCTCGTCGTCGCATTTCTCCA | Real-time PCR |
| *IbRAD18-F* | CCACCGTCTTCCTCTTCCTAC | Real-time PCR |
| *IbRAD18-R* | ACTCCCGAGTCTCCAATCAAC | Real-time PCR |
| *IbRD22-F* | AAGCACAGAGGTGTACGAGGTTT | Real-time PCR |
| *IbRD22-R* | AGTGGTTAGGGTTCCAGGCAGA | Real-time PCR |
| *IbActin-F* | GTTATGGTTGGGATGGGACA | Real-time PCR |
| *IbActin-R* | GTGCCTCGGTAAGAAGGACA | Real-time PCR |
| *AtActin2-F* | AGCACTTGCACCAAGCAGCATG | Real-time PCR |
| *AtActin2-R* | ACGATTCCTGGACCTGCCTCATC | Real-time PCR |

**Supplemental Table S1. Primers used for PCR analysis**
